# Understanding and Use of Nicotine Replacement Therapy and Nonpharmacologic Smoking Cessation Strategies Among Chinese and Vietnamese Smokers and Their Families

**DOI:** 10.5888/pcd11.130299

**Published:** 2014-02-20

**Authors:** Icarus K. Tsang, Janice Y. Tsoh, Ching Wong, Khanh Le, Joyce W. Cheng, Anthony N. Nguyen, Tung T. Nguyen, Stephen J. McPhee, Nancy J. Burke

**Affiliations:** Author Affiliations: Icarus K. Tsang, Ching Wong, Khanh Le, University of California, San Francisco, San Francisco, California; Tung T. Nguyen, Stephen J. McPhee, Nancy J. Burke, University of California, San Francisco, and Asian American Research Center on Health, San Francisco, California; Joyce W. Cheng, Chinese Community Health Resource Center, San Francisco, California; Anthony N. Nguyen, South East Asian Community Center, San Francisco, California.

## Abstract

**Introduction:**

Population-based studies have reported high rates of smoking prevalence among Chinese and Vietnamese American men. Although nicotine replacement therapy (NRT) is effective, recommended, and accessible without prescription, these populations underuse NRT for smoking cessation. The aim of this study was to assess understanding and use of NRT and nonpharmacologic treatments among Chinese and Vietnamese American male smokers and their families.

**Methods:**

In-depth qualitative interviews were conducted with 13 smoker–family pairs, followed by individual interviews with each participant. A total of 39 interviews were conducted in Vietnamese or Chinese, recorded, translated, and transcribed into English for analysis.

**Results:**

Four themes were identified: use and understanding of NRT, nonpharmacologic strategies, familial and religious approaches, and willpower. Both smokers and their family members believed strongly in willpower and a sense of personal responsibility as the primary drivers for stopping smoking. Lack of these 2 qualities keeps many Chinese and Vietnamese men from using NRT to quit smoking. Those who do use NRT often use it incorrectly, following their own preferences rather than product instructions.

**Conclusion:**

Our findings indicate the importance of culturally appropriate patient education about NRT. It may be necessary to teach smokers and their families at an individual level about NRT as a complementary approach that can strengthen their resolve to quit smoking. At a community level, public health education on the indication and appropriate use of evidence-based smoking cessation resources, such as NRT, would be an important component of effective tobacco control.

## Introduction

Chinese and Vietnamese Americans are 2 rapidly growing population groups in the United States (1). Disproportionately high rates of smoking prevalence have been reported among Chinese men (range, 22%–34%) (2–9) and Vietnamese men (25%–43%) with limited proficiency in English (9–12), compared with non-Hispanic white men (20.6%) (13). Evidence-based guidelines for smoking cessation recommend use of nicotine replacement therapy (NRT) (14). NRT in the forms of patch, gum, and lozenge are available in the United States without a physician’s prescription. Despite this, NRT remains underused (15–17), particularly among racial/ethnic minority populations such as Asian Americans. For example, population-based surveys showed that 83% of current Chinese smokers and 68% of current Vietnamese smokers quit without using smoking cessation aids (3,18). In minority communities, use of unassisted smoking cessation methods has been far more common than any assisted method (19). Thus, understanding factors that contribute to use of pharmacologic and nonpharmacologic assistance are essential to tobacco control endeavors. The aim of this study was to assess understanding and use of NRT and nonpharmacologic cessation methods from the perspectives of Chinese and Vietnamese smokers and their families.

## Methods

We conducted in-depth dyadic (paired) and individual interviews to inform the development of a family-based intervention focused on Chinese and Vietnamese smokers and their families (20). Eligible participants were aged 18 years or older, self-identified as Chinese or Vietnamese Americans, and Cantonese or Vietnamese speakers. Each dyad consisted of a smoker and a family member living in the same household at the time of the interview. Smoker participants had to have smoked at least 100 cigarettes in their lifetime and were either a current daily smoker (ie, smoked at least 1 cigarette daily in the past 7 days) or a former smoker (ie, had quit smoking within the past 5 years). A smoker participant could be self-referred or referred by the family member participant to the study and vice versa. Both the smoker and his family member participant had to agree to be interviewed together. Experience with quitting smoking or assisting a family member to quit smoking were not required or assessed before the interviews. A total of 13 smoker–family dyads were recruited through word-of-mouth and flyers posted at 3 community-based organizations located in San Francisco and San Jose, California. Research team members conducted interviews in either Cantonese or Vietnamese. The average length of the dyadic and individual interviews was 60 minutes; interviews were conducted about 2 to 3 weeks apart. Bilingual transcriptionists translated and transcribed each interview into English for analysis. This study was conducted with the approval of the University of California San Francisco Committee on Human Research.

We chose to conduct dyadic interviews primarily to gain insight into communication dynamics and smoking concerns within families and to observe nonverbal communication and relationship dynamics as they unfolded in the interview. Interviews started with a rapport-building discussion of participants’ backgrounds, including experiences in coming to the United States, living situation, and profession in country of origin. This led to a series of questions about smoking within the family: who smokes, how people feel about the smoking, conflicts that arise around smoking. We probed around these conflicts and communications to learn who was involved, the tone of discussions, and their outcomes. For dyads including a current smoker, we asked both participants to reflect on previous quit attempts, rationale for trying, what worked and what did not, who was involved, where participants found support, and who was not supportive. For dyads including a former smoker, we asked both participants to walk us through their quitting process and to discuss what they thought was most helpful in both the cessation (including NRT use) and maintenance phases of quitting. We also asked all participants to talk about how others in their families and broader social networks viewed the smoker’s behavior and, for former smokers, how quitting changed their family and broader social relationships. Following this discussion, we asked participants to look through a brochure and watch a public service announcement about the harms of smoking. Research team members left the room while the participants read through the brochure and viewed the public service announcement. After about 10 minutes, interviewers returned and asked participants about their reactions to the messages and relevance to their own experience.

Upon completion, translation, and transcription, 2 to 4 research team members read each dyadic interview transcript. On the basis of this reading and subsequent discussion, we developed a tailored individual interview guide for each participant. That is, while the dyadic interviews covered similar topics, the follow-up individual interviews focused on issues discussed in a particular dyadic pair’s interview. For example, in one dyadic interview, the wife discussed buying nicotine gum to support her husband’s quit attempts. In the follow-up individual interview, we learned that the smoker ignored the instructions included with the nicotine gum and used the gum only as needed.

The follow-up individual interviews were also translated into English and transcribed. To engage in collaborative analysis and to facilitate a “crystallization” process (21), we grouped transcripts together into families or sets (a set consisted of a dyad and 2 individual interviews) for analysis. First, we distributed sets to 4 research team members who were each asked to conduct a close reading and write a reflective summary of key findings from each set, new issues discussed, recurrent themes across interviews and interview sets, and data relevant to intervention development. At the same time, 2 team members coded each transcript by using a grounded theory approach (22). These team members individually coded the transcripts and met weekly to discuss codes and emergent themes. We used Atlas.ti qualitative software (ATLAS.ti GmbH, Berlin, Germany) to organize the coding and facilitate the combined analysis.

## Results

Participants included 8 current daily smokers, 5 former smokers, and 13 of their family members (1 for each smoker participant) aged from 19 to 65 years (median, 45.5 y). All smokers and family members were first-generation immigrants who had lived in the United States from 3 to 38 years (median, 7 y). Eleven of the 13 dyads included husbands (5 current and 6 former smokers) and their wives (10 never smokers, 1 former smoker). One dyad included a son (current smoker) and his mother (never smoker), and 1 dyad included a father (current smoker) and his son (never smoker).

Our collaborative and multilevel approach to analysis resulted in the identification of 4 primary themes related to smoking cessation. They are: 1) use and understanding of NRT, 2) nonpharmacologic strategies, 3) familial and religious approaches, and 4) willpower.

### Use and understanding of nicotine replacement therapy

Both smokers and family members had heard of NRT, which they referred to as “gum” (Cantonese, 香口膠; Vietnamese, kẹo cao su), “patch” (Cantonese, 貼片; Vietnamese, miếng dán nicotine), or “candy” (for lozenge) (Cantonese, 糖錠; Vietnamese, kẹo). In most instances, participants made no clear distinction between NRT gum or lozenges and regular gum or candy. However, when prompted, participants clarified that the terms given above actually referred to NRT gum and lozenges.

Both smokers and family members expressed mixed beliefs about the helpfulness of NRT; as one current smoker explicitly stated, “A lot of people use the patch or take medicines to help quit smoking. I didn’t use any of those, don’t know if they work. . . . I only ate candies or [salted] plums.” Another smoker felt that nicotine addiction could not be cured with medication: “No medicines can help to cure [nicotine] addiction. Chewing gums, eating plums, or snacks perhaps might help a little, but medicines are just impossible.” A wife of one smoker also conceived of smoking cessation as a gradual process and said, “‘Determination’ is more important than any NRTs. . . . Well, I advised him to quit but [his] smoking habit is heavy. It is only possible for him to quit gradually and not immediately. Quitting really requires one’s will and determination. . . . Nicotine gum? I am not sure it will work; I bought him plums and candies instead.”

Misconceptions about NRT were reported as obstacles that prevented smokers and family members from using and encouraging the use of these medications. For example, some smokers and family members raised concerns about the safety and side effects of NRT. One smoker expressed his concern: “I used [the nicotine gum and patch] separately. I was scared of the level of nicotine in my body.” Some smokers rejected using any kinds of pharmacologic aids: “I totally object to it! Medicine does have side effects. . . . I really don’t like to depend on medicine to help me quit.”

Even when NRT was used, smokers used NRT according to their own preferences, and instructions for proper use were not consistently followed. One former smoker said, “[Regarding] using the lozenges, according to the doctor, [they] must be used as described [in the instructions], but I used [them] differently. Whenever my mouth felt empty, then I took one.” A wife of a former smoker also stated, “Last New Year, he was given the nicotine patches [by his doctor] to use for a month, but he [only] used [the] nicotine patches for 6 to 7 days. [He] used [them] at the lowest dose.”

Chinese and Vietnamese smokers (current and former) and family members expressed hesitance about using NRT as a smoking cessation aid because of misconceptions of how NRT works and doubts of its effectiveness. Even among those who had used NRT (n = 4), participants deliberately chose not to adhere to the instructions for use and the treatment duration recommendation.

### Nonpharmacologic strategies: regular (nonnicotine) gum, candies, fruits, and salted plums

Participants reported using a variety of non-NRT aids — for example, some smokers mentioned using regular gum and candy (Cantonese, 普通香口膠/糖; Vietnamese, kẹo cao su/kẹo), terms to distinguish them from NRT gum and lozenges, in their quit attempts. Family members also spoke of buying gum and candies for the smokers in their family. Although smokers and family members reported some confusion about NRT gum and lozenges, they made a clear distinction about nonpharmacological aids by calling them regular gum and candy. They also mentioned using fruits and salted plums (common snacks eaten by Chinese and Vietnamese) during periods of craving for cigarettes.

Several smokers felt that these nonpharmacological aids were effective in reducing craving: “After a meal, it was [my] habit that I must go out in the yard to smoke [a cigarette]. . . . [If] I took a regular gum or candy to make me forget about the cigarette, then I saw it was effective.” A son of a smoker also bought plums and other snacks for his father to snack on weekly during his quitting; the son said, “Yes, some snacks for him like peanuts and plums, [I] bought for him [weekly].”

Both Chinese and Vietnamese smokers used these nonpharmacologic strategies to ease craving during quitting with the support from their family members.

### Familial and religious approaches

In addition to the use of nonpharmacological aids, smokers and family members reported that recollection of familial and filial duties helped bolster their commitment to quitting. One former smoker aimed to fulfill his familial and filial duties by quitting smoking: “[This is, I believe] a moral value. When you smoke, you’ll set a bad example. You think you can tell your children not to smoke later? No way. The second thing is about your parents. . . . If you smoke, you [are] not listening to their advice, that means you [do] not fulfill your filial duties.” Another wife of one smoker at times also communicated to her husband that her child’s health and her health would be affected if her husband continued to smoke: “Many times he tried to kiss my daughter but she said he smokes so much, [he] smells bad. She had that attitude [and it] made him embarrassed. So I told him he should quit for my daughter and my health. And I told him, ‘If you quit successfully, I will do whatever you want.’”

Chinese and Vietnamese smokers and their families cited cultural practices such as attention to religious observance as providing emotional support during the process of quitting. The wife of one smoker reported her husband listened to Buddhist chanting while quitting:

“After 1 week, 2 weeks, I was sure that he had quit smoking because I didn’t see him go outside; he stayed inside. He tried to control his urge [by] listening to Buddhist chanting.”

### Willpower

Smokers and family members repeatedly mentioned willpower or determination as being essential to smoking cessation. One former smoker reported, “In my case, after I came up with my decision and determination, I could quit easily.” Another smoker stated, “When I decide to quit smoking, my determination will surpass all. There is nothing more than [your] determination. . . . If you are not determined, you will deviate easily.” A wife of a former smoker also reported how she encouraged her husband by strengthening his determination: “Quitting really requires one’s will and determination. But the pressure that family has on him probably made him want to smoke more. So . . . in dealing with him . . . I try my best to help him quit [through] encouraging him and [strengthening] his determination.”

In many instances, willpower was thought to be more important than any other cessation aid. In fact, both smokers and their family members reported believing that willpower and determination might have kept smokers from trying pharmacologic cessation aids.

## Discussion

This qualitative investigation increases our understanding of Chinese and Vietnamese male smokers’ and their family members’ beliefs, experiences, and understanding regarding pharmacologic, nonpharmacologic, and unassisted smoking cessation strategies. Smokers and their family members shared many similar beliefs about the use of various smoking cessation strategies. All participants reported using nonevidence-based, nonpharmacologic smoking cessation strategies when quitting. In contrast, much fewer (n = 4) had used evidence-based cessation treatments such as quitline services and NRT. Indeed, only one smoker had heard about the California Smokers’ Helpline although the free quitline service has been available in Chinese and Vietnamese languages for more than 15 years (23). Our findings suggest that the belief about willpower, lack of awareness of available resources, and concerns about NRT side effects may underlie underuse of NRT and other evidence-based smoking cessation resources by Chinese and Vietnamese smokers.

Smokers’ concerns about NRT side effects, such as worries about the accumulation of nicotine in the body, have been reported in other studies with Chinese and Vietnamese smokers (19,24) and studies of other racial/ethnic groups (25). Only one of the former smokers received advice on NRT use from a physician; however, that person chose to use NRT according to his own preference but not the provider’s recommendation. Underuse and misuse of NRT has been identified recently as a major problem in smoking cessation in the general population (17). Addressing these concerns both in the clinical setting between provider and patients and in community-wide outreach would be an important part of a strategy to increase use of a proven smoking cessation method, particularly among underserved populations such as Chinese and Vietnamese Americans.

A prominent theme in the interviews was involvement of family members in the quitting process, and it was considered an essential part of the process by both smokers and their family members alike. Although this may have been biased by our selection criterion requiring both a smoker and a family member to participate, other studies have supported the importance of the family in the quitting process for Chinese and Vietnamese smokers. For example, among the Asian-language calls to the California Smokers’ Helpline, 40% were made by family or friends of smokers; only 6% of English-language calls were made by family or friends (23). Our findings suggest that clinicians should consider the role of family members and that public health outreach to address smoking cessation in these populations should focus on strategies to include family members and family health.

Consistent with previous studies (8,18), willpower was cited as a major determinant of successful quitting by both Chinese and Vietnamese smokers and their family members. Their belief that “willpower is all it takes” to quit smoking successfully might have discouraged smokers from using NRT or other evidence-based, nonpharmacological strategies. To encourage smokers’ use of evidence-based treatments, one approach that both clinicians and public health practitioners could take is to acknowledge the importance of willpower and to educate both smokers and their families about the use of evidence-based treatment as a complementary strategy that can further strengthen the willpower to quit smoking.

Our study had limitations. Our sample of Chinese and Vietnamese immigrant smokers and family members did not include American-born Chinese and Vietnamese, who may have different perceptions of quitting strategies. Our sample included only daily smokers or former smokers, and thus our findings may not apply to intermittent smokers. Typical of many qualitative studies, our study had some limitations because of its small sample size. For example, although our analyses identified many similarities among Chinese and Vietnamese smokers, we were unable to identify differences between the 2 Asian groups. Although the small sample size limits generalizability, this study used an established qualitative methodology that affords a deeper exploration of meaning and life circumstances in 2 Asian communities from the perspectives of both the smokers and their families. This more in-depth approach using both dyadic and follow-up tailored individual interviews offers a useful guide for the development of smoking cessation outreach programs that require attention to both individual and familial factors.

Studies have shown that two-thirds of US smokers quit without assistance (17). Effective strategies to promote appropriate use of evidence-based smoking cessation treatments at a community level, including NRT, remain unknown, particularly among underserved communities. By using in-depth interviews of Chinese and Vietnamese immigrant smokers and their family members, this study identified key barriers to underuse of NRT as over-reliance on willpower, misconceptions of how NRT works, and use of other nonevidence-based, nonpharmacologic methods. The findings provide insights into the concerns about NRT and understanding of its use that are shared by Chinese and Vietnamese smokers and their family members alike and that underlie its underuse. These insights may help clinicians and public health practitioners to create strategies to promote appropriate use of evidence-based treatment within the relevant cultural beliefs and contexts for these populations, such as the importance of including family support and the use of NRT to supplement willpower.
